# Biomarker-Based Approaches for Assessing Alcohol Use Disorders

**DOI:** 10.3390/ijerph13020166

**Published:** 2016-01-27

**Authors:** Onni Niemelä

**Affiliations:** Department of Laboratory Medicine and Medical Research Unit, Seinäjoki Central Hospital and University of Tampere, Seinäjoki 60220, Finland; onni.niemela@epshp.fi; Tel.: +358-6-4154719; Fax: +358-6-4154924

**Keywords:** ethanol, health, aminotransferase, GGT, CDT, fibrosis, NASH, obesity, oxidative stress

## Abstract

Although alcohol use disorders rank among the leading public health problems worldwide, hazardous drinking practices and associated morbidity continue to remain underdiagnosed. It is postulated here that a more systematic use of biomarkers improves the detection of the specific role of alcohol abuse behind poor health. Interventions should be initiated by obtaining information on the actual amounts of recent alcohol consumption through questionnaires and measurements of ethanol and its specific metabolites, such as ethyl glucuronide. Carbohydrate-deficient transferrin is a valuable tool for assessing chronic heavy drinking. Activities of common liver enzymes can be used for screening ethanol-induced liver dysfunction and to provide information on the risk of co-morbidities including insulin resistance, metabolic syndrome and vascular diseases. Conventional biomarkers supplemented with indices of immune activation and fibrogenesis can help to assess the severity and prognosis of ethanol-induced tissue damage. Many ethanol-sensitive biomarkers respond to the status of oxidative stress, and their levels are modulated by factors of life style, including weight gain, physical exercise or coffee consumption in an age- and gender-dependent manner. Therefore, further attention should be paid to defining safe limits of ethanol intake in various demographic categories and establishing common reference intervals for biomarkers of alcohol use disorders.

## 1. Introduction

Alcohol use disorders, both acute and chronic, are significant clinical problems due to their devastating health impacts and high prevalence throughout the world [1,2,3,4,5]. Virtually all tissues in the body can be affected by excessive alcohol consumption and a wide variety of alcohol-related disorders are currently known. For successful clinical interventions, hazardous drinking should be detected in an early phase to prevent the affected individuals from entering a stage of severe dependence with associated tissue toxicity. 

The occurrence of health problems in alcohol consumers seems to be proportional to the amount of alcohol ingested over a long period of time [1,2,3,4]. Chronic alcohol drinking exceeding 300 g (men) or 200 g (women) per week is known to sharply increase the risk for damage [6,7]. In women, adverse effects may arise at lower levels and alcohol-related problems concerning pregnancy could add another dimension to the problem of excessive alcohol consumption *per se* [3,4,8]. In individuals with risk factors such as obesity, smoking or hepatitis C infection, health problems can also be triggered by relatively low levels of alcohol intake [9,10,11,12,13,14]. Recent American Association for the Study of Liver Diseases (AASLD) guidelines on non-alcoholic fatty liver disease (NAFLD) defined alcohol consumption exceeding 21 drinks (~250 g) per week in men and 14 drinks (~170 g) per week in women as limits of significant alcohol consumption [15]. However, current lifetime risk evaluations have indicated that even levels of 14 drinks per week for men or seven drinks per week for women can increase alcohol-attributable mortality [16].

Recent developments in the treatment of patients with alcohol use disorders have emphasized the role of biomarkers as an integral part of the assessment [17,18,19,20,21]. Biomarkers are markers of a biological process or state, which are useful for clinicians and patients if they provide information about the current status or future risk of disease [22]. In alcohol use disorders, biomarkers should be used not only to confirm the aetiology but also to help the interactions between physicians and patients on raising the issue of alcohol use as a possible cause of adverse health outcomes. They can also improve patient follow-up procedures providing useful prognostic information. Biomarker-based evaluations may also open new insights on the primary mechanisms of ethanol-induced diseases. The aim of the present contribution is to discuss the current role of biomarkers in the assessment of alcohol consumption and associated health problems. For additional information, the reader is referred to other previous reviews in this field [17,18,19,20,21,23].

## 2. Biomarkers of Alcohol Consumption *per se*

Both the amounts and patterns of ethanol consumption determine the risk of developing alcohol addiction and associated morbidity. Information on the actual amounts of alcohol consumption can be collected by specifically designed questionnaires such as Alcohol Use Disorders Identification Test (AUDIT), CAGE alcohol questionnaire (Cut down, Annoyed, Guilty, Eye-opener), Michigan Alcoholism Screening Test (MAST) or time-line follow-back (TLFB) [24,25]. While the first three are screening tools covering various aspects of alcohol consumption, problems and dependency, TLFB calendar assessment provides estimates of the actual amounts of consumption. All of these are, however, dependent on self-reports, which are memory-dependent and often unreliable channels of information. Prevailing attitudes towards drinking both among patients and health care personnel can also influence the outcome of the questionnaires in clinical settings. Therefore, laboratory tests are often needed to provide additive information (Table 1). 

Measurements of ethanol itself reveal ethanol intoxication. They can also be used in the assessment of compliance during treatment [17]. In alcohol-dependent patients, positive blood ethanol may be seen even at the time of the clinic visit. Based on blood ethanol findings and clinical observations it is possible to reach conclusions on long-term drinking habits. Ethanol levels exceeding 1.5‰ (33 mmol/L) without any apparent signs of intoxication indicate ethanol tolerance, which is a typical sign among alcohol-dependent individuals. In fact, in health care settings, the occurrence of positive blood alcohol levels at any time should lead to a suspicion of heavy drinking history [26]. 

The short half-life of ethanol often prevents physicians from routinely ordering these tests. Ethyl glucuronide (EtG), a minor nonoxidative metabolite of ethanol, is formed in the liver by enzymatic conjugation of ethanol with glucuronic acid and this metabolite can be analyzed by immunological or liquid chromatograpy-mass spectrometry techniques from different types of biological fluids, hair or nails [27,28,29,30,31,32]. Depending on the sample type used, EtG may remain positive for several days after cessation of ethanol intake and it can thereby provide additional value when assessing recent alcohol consumption [28]. Studies so far have indicated useful diagnostic applications for EtG in post-mortem evaluations of alcohol drinking [30], assessment of fetal alcohol exposure [27,29,33,34] or in patients scheduled for liver transplantation [35]. Ethyl sulfate (EtS) is another conjugated metabolite of ethanol, which is formed in low amounts after alcohol consumption [28,36]. Monitoring both EtG and EtS is, however, usually unnecessary [37]. Phosphatidylethanol (PEth) is a specific long half-life metabolite of ethanol, which is formed in the body only when ethanol is present. This phospholipid species increases in a highly sensitive manner in biological fluids as a consequence of alcohol drinking [38,39,40,41]. Stability of PEth has recently shown to be good in assays from dry blood spot cards, which may further improve the potential of PEth for routine applications [41]. Fatty acid ethyl esters (FAEE) are formed by esterification of ethanol with free fatty acids [42]. Assays of FAEE by gas chromatography-mass spectrometry techniques from hair have been suggested as possible tools for retrospective detection of alcohol abuse during pregnancy or in forensic applications [33,34,42]. Acetaldehyde is the first metabolite of ethanol, which, due to its high reactivity, is capable of binding to proteins and cellular constituents during ethanol metabolism [43,44]. Such binding creates distinct neoantigenic epitopes and immune responses, which have been suggested not only as diagnostic tools but also as an important pathogenic feature underlying alcohol-induced tissue toxicity [43,44,45,46].

**Table 1:** Biomarkers of alcohol consumption.

Biomarker	Abbreviation	Biological Sample Type	Marker Characteristics
Ethanol	EtOH	Blood Urine Breath	Restricted to conditions where ethanol is still present in circulation.
Ethyl glucuronide /Ethyl sulfate	EtG/EtS	Urine Serum Cerebrospinal fluid Vitreous humour Hair Nails	Ethanol metabolite, which remains positive in urine samples 2–5 days after stopping ethanol use. Window of detection dependent on sample type.
Phosphatidylethanol	PEth	Blood Dry blood spots	Ethanol metabolite, which remains detectable 1–2 weeks after alcohol use. Measured by LC-MS or immunological techniques.
Fatty acid ethyl esters	FAEE	Plasma Hair Meconium	Ethanol metabolite derived from a combination of fatty acid with alcohol.
Acetaldehyde adducts and associated immune responses	AA-Ab	Blood Tissue specimens	IgA response towards acetaldehyde adducts most specific for alcohol-related disorders.
Carbohydrate-deficient transferrin	CDT	Serum Cerebrospinal fluid	Specific marker of chronic alcohol consumption. Lacks sensitivity for screening purposes.
Gamma-glutamyltransferase	GGT	Serum/plasma	Sensitive marker of alcohol use, liver dysfunction and oxidative stress. Several sources of unspecificity. Normalization time 2–3 weeks.
GGT-CDT combination	GGT-CDT	Serum/plasma	Improves sensitivity and specificity of detecting alcohol abuse. Relies on a mathematical model.
Blood cell counts		Blood	Mean corpuscular volume (MCV) of erythrocytes typically elevated in alcoholics. Normalization time 2–4 months. Mean corpuscular haemoglobin (MCH) and thrombocytes (platelet counts) are also frequently altered in alcohol abusers. Several sources of unspecificity.
Transaminase enzymes	ALT, AST	Serum/plasma	Suitable for screening for liver dysfunction in alcohol users. Sensitive to effects of excess body weight. AST/ALT ratio increases in alcoholic liver disease.

Elevated levels of serum carbohydrate-deficient transferrin (CDT) reveal chronic alcohol abuse in a rather specific manner (Table 1). Both the amounts of disialo- and asialo-isoforms of transferrin increase as a result of heavy alcohol intake and this abnormal sialylation pattern can be analyzed by immunological techniques, high performance liquid chromatography or capillary electrophoresis [18,47,48]. Interestingly, the levels of total serum sialic acid also increase in association with glycoprotein desialylation as a result of heavy alcohol intake [49,50]. Unlike many other biomarkers, CDT is more sensitive to changes in ethanol consumption than to the secondary effects of liver disease, and it can also help to differentiate between alcoholic *versus* non-alcoholic liver disease. However, it should be noted that CDT assays, which are sensitive to changes in serum total transferrin, also fluctuate in response to the status of liver disease *per se* [51]. CDT elevations require consumption of at least 50–80 g of ethanol per day for a period of several weeks and, thus, it lacks sensitivity as a screening tool in general populations. In alcohol-dependent patients, it is, however, sensitive enough for detecting relapses and monitoring sobriety [48,52,53,54].

Gamma-glutamyltransferase (GGT) is a membrane-bound glycoprotein enzyme, which has long been used as a marker of excessive alcohol intake (Table 1) [55,56]. GGT is sensitive to changes in alcohol consumption, but, due to lack of specificity, it is not suitable for screening among populations with non-alcoholic liver diseases, obesity or hospitalized patients [17,57]. In alcoholics, increased activities usually return to normal within 2–3 weeks upon abstinence, whereas persistently abnormal values may suggest liver disease. 

Previous work has indicated that diagnostic improvement in detecting alcohol use disorders could be achieved by combining two or more alcohol markers [17,21]. The conventional manner of combining markers is to see whether either is elevated [48,58]. This approach obviously gives improved assay sensitivity but is frequently associated with a decrease in specificity. However, combination of GGT and CDT using a mathematically formulated equation GGT-CDT = 0.8 × ln(GGT) + 1.3 × ln(CDT) can improve the detection of excessive alcohol consumption by increasing assay sensitivity without a loss in specificity [58]. This marker is elevated in a higher percentage of alcohol abusers than either GGT or CDT alone and reacts after regular ethanol consumption exceeds a threshold of 40g per day. The correlations with the actual amounts of ethanol consumption and GGT-CDT are also higher than those of its parent components [58]. 

Hazardous drinking practices also create typical abnormalities on blood cell counts and their morphological features, particularly on erythrocyte and thrombocyte lineages (Table 1) [59]. There seems to be a dose-dependent response between erythrocyte size (mean corpuscular volume, MCV) and ethanol intake [60]. Mean corpuscular haemoglobin (MCH) is also elevated in heavy drinkers. Upon abstinence, normalization of red cell indices may require 2–4 months. In heavy drinkers without co-morbidities, high MCV values are typically seen without anaemia, whereas in patients with alcoholic liver disease and a concomitant folate deficiency, megaloblastic bone marrow alterations and haemolysis, high MCV and anaemia usually co-exist [61]. Erythrocytes from alcoholics are prone to damage and shortened biological half-life, which may be associated with modifications of proteins and cell membrane constituents by acetaldehyde and reactive aldehydic products of lipid peroxidation [44,59]. Blood platelet counts are decreased in one third of the alcoholics [61]. Upon abstinence, the levels return to normal usually within a few days. A low thrombocyte count associated with increased liver transaminase (aspartate aminotransferase, AST, and alanine aminotransferase, ALT) enzymes—and possibly increased AST/ALT ratio—can be considered an early warning sign of developing alcoholic liver disease.

A wide variety of other laboratory markers are also altered in response to excessive alcohol use, although without sufficient specificity to serve as biomarkers of alcohol abuse. Heavy alcohol consumption increases serum uric acid, a compound with free radical scavenging properties, which may indicate an increased need for antioxidant capacity under such conditions [62,63,64]. Uric acid also correlates with the activities of liver enzymes in alcohol consumers [62]. In lipid profiles from heavy drinkers, increased high density lipoprotein-cholesterol (HDL) is observed even following regular alcohol intake of less than five drinks per day. Excess drinking also frequently leads to dysregulated fat metabolism, as reflected in increased levels of serum triglyserides and free fatty acid ethyl esters. Such findings also associate with increased hepatic fat content, glucose dysregulation, and low-grade inflammation [65].

## 3. Liver Enzymes as Indicators of Hepatic and Extrahepatic Effects of Alcohol

The liver is a major target of ethanol toxicity due to its primary role in ethanol metabolism [2,3,4]. Therefore, unexpected abnormalities in liver enzyme activities, GGT or ALT, are frequently the first clinical signs of excessive alcohol consumption. Measurements of these enzymes are also widely used as screening tools for abnormal liver function and in decisions to select patients needing the closest monitoring. 

Fatty liver disease associated with obesity (NAFLD) is the most common non-alcoholic cause of increased GGT and ALT activities [14,15,66,67,68,69]. Alcohol use and obesity often co-exist and create toxicity in a synergistic manner [9,69,70,71,72]. Alcoholic liver disease (ALD) and NAFLD can also be overlapping phenomena and the threshold levels of harmful alcohol consumption in individuals with varying body weights have not yet been established. In obese persons, increased GGT and tissue morphology similar to alcohol excess is common even in those drinking an average of two drinks per day [9]. This may be explained by induction of common pathways of oxidative stress since GGT plays a key role in the metabolism of glutathione (GSH) and in the regulation of oxidative stress [9,13,73,74,75,76,77,78]. GGT could also be interpreted as a biomarker of oxidative stress indicating an increased need to maintain intracellular GSH levels [73,79,80].

Interestingly, in current populations, there seems to be a trend even towards permanent GGT increases [79]. Studies have further shown an association between GGT levels and a variety of extrahepatic chronic diseases, which are associated with oxidative stress, including cardiovascular diseases, diabetes, metabolic syndrome, cancer, neurodegenerative diseases and rheumatoid arthritis [81,82,83,84,85,86,87]. While the specific role of alcohol as a possible trigger for such morbidity has remained unknown, it should be noted that recent studies have indicated that even light to moderate alcohol drinking can lead to an elevated risk of cancer [88] and an increase in all-cause mortality [16,89]. Elevated GGT is associated with increased cardiovascular risk especially in men with simultaneous evidence of hepatic steatosis [90,91,92,93]. Furthermore, recent studies have linked the development of fatty liver and early atherosclerosis with the ability of GGT to trigger iron-dependent oxidation of low density lipoprotein (LDL) in coronary plaques [94]. Studies have also noted significant correlations between LDL-cholesterol and GGT levels, especially in men [95]. However, GGT levels are also associated with mortality outcomes independently of fatty liver [87].

Alcohol abuse is also a common cause of increased serum aminotransferase (ALT,AST) activities. ALT originates primarily from the hepatocytes, whereas AST is also abundant in heart, skeletal muscle tissue, kidneys, and the brain. Thus, serum ALT has been considered a more specific marker of liver affection, whereas AST often shows increased activities due to extrahepatic reasons, including muscle diseases or strenuous exercise [96]. Current estimates have indicated that over half of the aminotransferase abnormalities in Western countries result from obesity and related comorbidities [14,97,98]. The occurrence of alcohol consumption and adiposity together also increases the risk of abnormal transaminase activities and while GGT enzyme seems to be relatively more sensitive to ethanol intake, ALT may be the predominant responder towards increasing BMI [9,71,99]. In obesity, ALT activities correlate with ectopic fat deposition, and the values decline with weight loss [100,101]. Increased ALT levels are also linked with extrahepatic health risks, such as type 2 diabetes, metabolic syndrome, and insulin resistance [72,83,102,103,104]. They also predict vascular morbidity [72,92,102,103,104,105,106,107,108]. 

When interpreted together, aminotransferases can provide information on the nature of liver dysfunction. The elevation of the AST/ALT ratio over one has been considered suggestive of alcoholic aetiology [96,109,110,111]. Such findings may be explained by depletion of pyridoxine (B6) vitamin for ALT biosynthesis, more pronounced hepatic mitochondrial damage or skeletal or cardiac muscle injury (alcoholic myopathy), which release AST into circulation [109,112]. Elevated AST/ALT ratios have, however, also been reported from non-alcoholic steatohepatitis (NASH) patients with a high fibrosis risk [55,113,114]. 

## 4. Impacts of Gender, Age and Life Style

**Figure 1:**
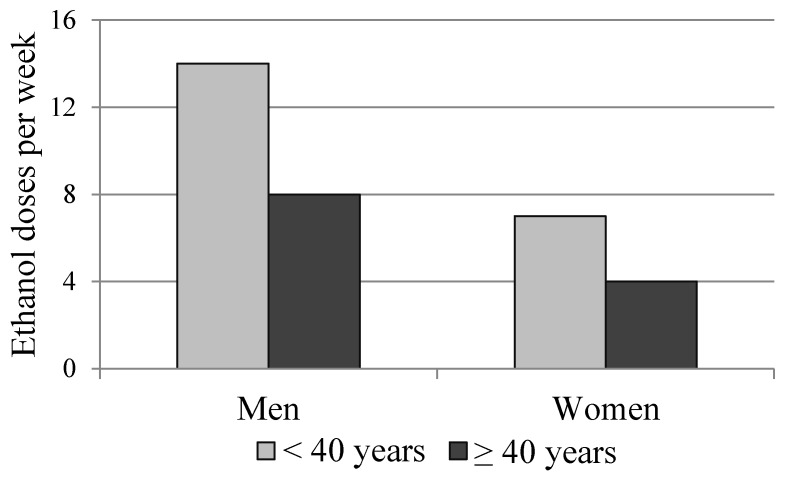
Threshold levels of alcohol consumption (standard drink units/week) for initiating GGT activation in individuals below and above 40 years of age. Alcohol consumption was recorded from the past one year prior to sampling [116]. The levels leading to GGT increases are markedly lower than the current limits of heavy drinking in many Western countries (men: 24 drinks, women: 16 drinks).

Many ethanol-induced biochemical changes take place in a gender-dependent manner [3,9,62,95,115]. The individual susceptibility to disorders such as liver cirrhosis, brain damage, heart disease or alcohol-induced cancer is markedly higher in women despite the fact that women generally drink less alcohol over their lifetime [3,88,115]. Lower limits for safe drinking levels are also recommended for women [16]. Women have less water in their body and therefore it is believed that women are exposed to higher concentrations of alcohol and its toxic metabolites during periods of alcohol drinking and ethanol metabolism. In women, GGT levels are also elevated after ingestion of lower levels of alcohol than in men (Figure 1).

Recent studies have also emphasized increasing age as an important determinant of alcohol-related toxicity. In individuals over 40 years of age only eight standard drinks for men and four drinks for women as levels of regular ethanol consumption per week lead to first signs of GGT activation (Figure 1). Although in those below 40 years old the corresponding threshold doses are higher, it should be noted that both levels of consumption are clearly lower than the currently used limits of heavy drinking in many countries. Since recent population studies have emphasized high mortality rates among older individuals consuming alcohol [117], the concept of safe limits for ethanol intake should obviously be revisited not only between genders but also among different age categories. In addition, in experimental animals, aging has been shown to promote the development of diet-induced steatohepatitis and induction of liver enzyme levels [118].

The composition of the diet and the presence or absence of obesity are important co-factors in determining body responses to alcohol consumption [9,11,71,119]. Induction of liver enzyme activities together with elevated blood lipid levels may be seen even among young individuals with overconsumption of the Western diet [95,118]. In experimental animals, adverse effects of ethanol are aggravated by high-fat-diets [120] or diets deficient in folate [121]. Excess dietary iron also exacerbates ethanol toxicity [120]. Genetic variation in adiponutrin (PNPLA3) or in alcohol-metabolizing enzymes also seem to play a role in conferring susceptibility to tissue damage [14]. 

Recent studies have further indicated a synergistic toxic effect of smoking on ethanol-induced liver pathology and activation of GGT enzyme [10,122]. On the contrary, in heavy drinkers with regular coffee consumption, GGT levels seem to be relatively lower than in heavy drinkers without any coffee consumption, indicating a possible protective effect of coffee towards alcohol-induced liver damage and associated oxidative stress [123,124,125]. Coffee consumption seems to modulate the effect of ethanol in a dose- and gender-dependent manner, the most striking effects being found among men who drink over four cups of coffee per day [123]. Regular aerobic exercise reduces hepatic lipids even in the absence of body weight reduction and could also provide protection towards oxidative stress [126,127].

## 5. Differential Diagnosis of Alcoholic *versus* Non-Alcoholic Causes of Tissue Toxicity

Clinical symptoms of alcohol toxicity are often unspecific and may arise from virtually any tissue [3,6,18,128]. Alcohol-consuming patients, however, tend to escape specific treatment because the clinicians ability to detect alcohol abuse is often constrained by the difficulties in obtaining reliable reports on alcohol intake [19]. In addition to obtaining information on current drinking habits by specific questionnaires and biomarkers, a wide selection of biomarkers is also available to rule out possible non-alcoholic etiologies (Table 2). For example, in patients with suspected liver affection, NAFLD is known to be the most common non-alcoholic etiology and evaluation of metabolic co-morbidities with measurements of body mass index, waist circumference, and oral glucose tolerance are helpful [14,18]. Many competing and co-existing causes of abnormal liver function can be excluded by appropriate serological and genetic tests (Table 2). In a similar manner, combined use of tissue-specific laboratory markers with markers of ethanol consumption, such as CDT, can be used to detect the possible alcoholic origin in pancreatic disorders [129].

**Table 2:** Biomarker-based differential diagnosis of abnormal liver function.

Condition	Supporting Laboratory Data	Other Diagnostic Tools
Fatty liver		
Alcoholic	Alcohol, EtG, GT, CDT, ALT, AST, MCV	Questionnaires: AUDIT, TLFB, CAGE, MAST
Non-alcoholic (obesity)	ALT, AST, glucose, OGT, triglycerides, PNPLA3 genotyping	BMI, waist circumference, abdominal ultrasonography
Viral hepatitis	A: anti-HAV IgM; B: HBsAg, PCR, anti-HBc IgM; C: anti-HCV, PCR; D: anti-HDV; E: anti-HEV; G: anti-HGV	
Liver cirrhosis	Albumin, bilirubin, prothrombin time, immunoglobulins, markers of immune activation and fibrogenesis	Liver biopsy, xenobiotic metabolism and excretion tests, liver imaging: ultrasound, MRI, Fibroscan, measures of hepatic function: Child-Pugh, CCLI, CMI
Drug toxicity	Transaminases, therapeutic drug monitoring, blood eosinophils	Case history
Hemochromatosis	Iron status, transferrin iron saturation, ferritin, HFE-genotyping (C282Y mutation)	Liver biopsy (hepatic iron index)
Autoimmune diseases		
Autoimmune hepatitis	Immunoglobulins, antinuclear antibodies, antismooth muscle antigen	
Primary biliary cirrhosis	AP, IgM, antimitochondrial antibodies	
Primary sclerosing cholangitis	ANCA, AP	ERCP
α1-antitrypsin deficiency	α-1-antitrypsin phenotyping	
Wilson’s disease	Ceruloplasmin, urine and hepatic copper	
Celiac disease	Tissue transglutaminase antibodies	
Strenuous exercise	AST, ALT, myoglobin, creatinine kinase	
Malignant condition	AFP	Ultrasound
Idiopathic	Absence of markers	Liver biopsy

ALT: alanine aminotransferase; ANCA: anti-neutrophil cytoplasmic antibody; AP: alkaline phosphatase; AST: aspartate aminotransferase; AUDIT: alcohol use disorders identification test; BMI: body mass index; CAGE: alcohol questionnaire; CDT: carbohydrate-deficient transferrin; ERCP, endoscopic retrograde cholangiopancreatography; EtG: ethyl glucuronide; GGT: gamma-glutamyltransferase; MAST; Michigan alcoholism screening test; MCV, mean corpuscular volume of erythrocytes; OGT: oral glucose tolerance; PCR, polymerase chain reaction; PNPLA3: patatin like phospholipase-3; TLFB: time line follow-back.

## 6. Markers of Disease Prognosis

Scoring systems based on selected combinations of biomarkers have been developed for assessing severity of alcohol-induced tissue damage. In patients with liver disease, algorithms such as the Child–Turcotte–Pugh score, Model for End-Stage Liver Disease, and Combined Clinical and Laboratory Index reflect overall liver function, life expectancy and surgical mortality [19,130] (Table 3). These parameters correlate with disease prognosis and help to stratify expected disease outcome and to identify high-risk patients for therapy. The laboratory indices selected in these models also show significant correlations with important morphological indices of disease severity, such as combined morphological index (CMI) [130].

**Table 3:** Biomarker-based scoring systems for the severity of alcoholic liver disease.

Score	Full Name	Clinical and Histological Components	Laboratory Components
CPT	Child-Pugh-Turcotte	Ascites, encephalopathy	Albumin, bilirubin, prothrombin time
MELD	Model of end-stage liver disease		Bilirubin, creatinine, INR
MDF	Maddrey discriminant function		Bilirubin, prothrombin time
GAH	Glascow alcoholic hepatitis score	Age	White blood cell count, urea, prothrombin time, bilirubin
CCLI	Combined clinical and laboratory index	Ascites, encephalopathy, collateral circulation, edema	Hemoglobin, albumin, bilirubin, alkaline phosphatase, prothrombin time
CMI	Combined morphological index	Necrosis, inflammation, cMallory bodies	Correlates with laboratory indices of prognostic significance

Among the most high-impact biomarkers for assessing the severity of alcoholic liver disease (ALD) are serum bilirubin and liver-derived proteins. Bilirubin is an insoluble breakdown product of heme, which is conjugated to glucuronic acid in the liver [55]. Strongly (5–10 fold) elevated bilirubin levels have been shown to be a highly significant prognostic determinant and is included in most algorithms (Table 3) [130]. Concentrations of serum albumin, ferritin, and blood clotting factors also show characteristic changes in response to liver disease stage [55]. The half-life of albumin is about 20 days, whereas that of clotting factors is only about one day. Serum albumin, which also plays a functional role as a circulating antioxidant, is often slightly elevated in heavy drinkers devoid of liver disease [131,132]. In patients with advanced liver disease protein synthesis rates are markedly decreased and levels below 25 g/L associate with poor prognosis [55,133]. In alcohol consumers without apparent liver disease, serum ferritin synthesis rates are also increased, which can be associated with disturbances in cellular iron homeostasis and the risk of secondary iron overload [133,134]. Iron and alcohol can also act in a synergistic manner to enhance lipid peroxidation, oxidative stress and associated liver injury [12,120,135,136]. On the other hand, serum ferritin can sequester catalytically active free iron, which has been considered a possible defense mechanism towards ethanol-induced oxidative stress [137]. 

## 7. Biomarkers of Fibrogenesis

Fibrosis in alcoholics is a response to injury, cell death and inflammation, constituting a major determinant of patient outcome [20,138,139]. Although progression of fibrosis to irreversible cirrhosis is largely dependent on the amounts of alcohol consumed over a long period of time, it may also occur in an unpredictable manner in susceptible individuals. The gold standard of diagnostics is the morphological examination of biopsy specimens, which is, however, a costly and invasive approach with a possible risk of complications. Therefore, biomarkers for following the activity of excess connective tissue deposition are also required. Over the past decades, several non-invasive tools have been introduced to allow repeated examinations during patient follow-up (Table 4). In addition to specifically designed imaging techniques (Fibroscan), biomarkers based on collagen type-specific peptides and various laboratory algorithms have become available [18,20,140]. 

Type I and type III collagens are the main types of collagen accumulating in hepatic tissue in response to alcoholic injury. The latter is more pliable and therefore type III procollagen derived fragments have been preferred as biomarkers [18,141]. The aminoterminal propeptide of type III procollagen (PIIINP), is elevated in ALD and the measurements help to identify patients with progressive collagen deposition [141]. Hyaluronic acid (HA), a mucopolysaccharide synthesized by fibroblasts and hepatic stellate cells, also increase in ALD correlating with the progression of perisinusoidal fibrosis and cirrhosis [138]. 

The inability of collagen degradation to keep pace with increased biosynthesis is a typical feature of progressive fibrosis. The degradation of extracellular matrix is regulated by tissue inhibitors of metalloproteinases (TIMPs), which are usually elevated in alcoholics with precirrhotic states [138,139]. In severe stages of ALD, there seems to be prominent elevations in serum PIIINP and proinflammatory cytokines (IL-2, IL-6, IL-8, TNF-α), which coincides with low levels of markers of fibrolysis and anti-inflammatory cytokines (IL-10, TGF-β) [142,143]. Assays reflecting the disturbed balance between collagen synthesis and degradation have been proposed to provide more accurate estimates of the collagen deposition rates than analyses of any single connective-tissue derived peptide [20,138,139,140,144,145] (Table 4). At this time, Fibrotest is the most widely used such algorithm in Europe [140]. ELF (Enhanced Liver Fibrosis), a test combining serum PIIINP, hyaluronic acid and TIMP, has also shown significant correlations with histological findings in the follow-up of fibrogenesis [146]. Other markers include combinations of connective tissue components with blood platelet levels [147] (Table 4).

**Table 4:** Biomarkers of fibrogenesis.

Marker	Abbreviation	Components in Combination
Connective tissue derived peptides		
Aminopropeptide of procollagen type III	PIIINP	
Aminopropeptide of procollagen type I	PINP	
Carboxypropeptide of procollagen type I	PICP	
Carboxyterminal telopeptide of type I collagen	ICTP	
Hyaluronic acid	HA	
β-Crosslaps	β-CTX	
Tissue inhibitor of matrix metalloproteinase	TIMP	
Combination markers		
Fibrotest		GGT, ALT, α-2-macroglobulin, haptoglobin, apo A1, bilirubin
Enhanced liver fibrosis	ELF	PIIINP, hyaluronic acid, TIMP
AST/platelet ratio	APRI	AST, platelet count
Traffic light test	TLT	PIIINP, hyaluronic acid, thrombocytes

## 8. Markers of Immune Activation in Alcohol Use Disorders

Table 5 summarizes useful conventional and novel biomarkers of immune activation in alcoholic patients. The presence or absence of inflammation is a key determinant of patient outcome in the pathogenesis of alcohol use disorders. In alcoholic liver disease, an altered balance between pro- and anti-inflammatory status is related with progression of fibrogenesis. Proteins expressed by immunologically active cells, such as soluble urokinase plasminogen activator receptor (suPAR) is increased as a result of heavy alcohol consumption and further with the development of liver disease [148]. Several lines of recent evidence have shown that CD163, a biomarker reflecting the activity of Kupffer cells, yields prognostically important information in alcoholic patients [149,150]. CD163 is an endocytic receptor for haptoglobin-hemoglobin complexes and is expressed specifically on macrophages and monocytes. This biomarker also seems to show potential to identify those at risk of developing liver cirrhosis [149,150,151]. Progression of liver damage in alcohol abusers is also associated with the generation of specific immune responses directed towards chemical modification of proteins by acetaldehyde [44,152,153]. 

Conventional biomarkers of inflammation, including high sensitivity CRP, and proinflammatory cytokines, such as IL-6, can also contribute to the assessment of changes occurring even in the central nervous system in alcohol abusers [154,155]. The presence or absence of inflammation seems to play a pivotal role in alcohol-induced mental disorders and depression such that patients presenting with a pro-inflammatory status may be expected to be more resistant to treatment efforts.

## 9. Reference Values for Biomarkers 

An ideal biomarker for identifying alcohol use disorders should be easily measurable, accurate, reproducible, cost-effective and easy to interpret by the clinician [22]. Biomarkers should help to separate patients with the disease state from the individuals who are in good health. Biomarker-based approaches for assessing alcohol use disorders at this time are, however, far from ideal due to lack of knowledge concerning the definition of biomarker reference intervals [156,157,158,159]. Although an extensive amount of previous literature is available on biomarkers of alcohol consumption, the information on the sensitivities and specificities of even the most commonly used markers has remained controversial. Many marker studies have contrasted extreme populations such as obvious alcoholics to teetotallers. Studies may also have failed to distinguish between the amount of alcohol consumed and the secondary effects of liver disease. On the other hand, studies aimed at establishing biomarker normal limits may have failed to exclude individuals who exceed the limits of consumption which—in light of current data—are associated with increased health risks. Not surprisingly, the upper normal limits even for the most common liver enzymes show a great deal of variation between individual laboratories as well as between different countries [95,157,158]. The differences are especially striking in those markers, which are most sensitive to ethanol consumption and obesity-related morbidity, such as ALT and GGT. 

**Table 5:** Biomarkers of immune activation in alcoholics.

Marker	Abbreviations	Characteristics
Macrophage receptor for haptoglobin-hemoglobin complexes	CD163	Marks Kupffer cell activation. Elevated levels are associated with poor prognosis.
Soluble urokinase plasminogen activator receptor	suPAR	Marks activation of inflammatory cells. Associated with disease severity.
Cytokines Proinflammatory Anti-inflammatory	TNF-α, IL-6, IL-8 IL-10, TGF-β	An altered balance in the ratio of proinflammatory and anti-inflammatory cytokines is typical during the course of liver disease progression in alcoholics.
Immune responses towards ethanol metabolites	Anti-acetaldehyde adduct IgA, IgG, IgM	Anti-adduct IgAs are typical in ALD. Useful for differential diagnosis between alcoholic and non-alcoholic causes of liver disease.
High sensitivity C-reactive protein	hs-CRP	A marker of low-grade-inflammation. Associated with pro-inflammatory status, which also contributes to multiple alcohol-induced mood disorders, including depression.

Recent surveys have indicated that if the reference populations consist of typical apparently healthy individuals with a wide range of body mass index and alcohol consumption up to 20 standard drinks per week, the upper normal limits computed based on such populations would become 29%–40% and 12%–92% higher than the corresponding limits based on normal weight abstainers for ALT and GGT, respectively (Table 6). It is obvious that the concept of normal limits for any biomarker sensitive to alcohol consumption needs to be revisited in different demographic populations and over a range of different ages. This is also an important prerequisite for successful implementation of early intervention programs. 

**Table 6:** Comparison of upper limits of normal (ULN) of two liver enzymes based on two different types of reference populations.

Liver Enzyme	Reference Population		
	Normal Weight Non-Drinkers	Moderate Drinkers with or without Overweight	Difference
ALT (U/L)			
Men	50	70	+40%
Women	35	45	+29%
GGT (U/L)			
Men	60	80 (age < 40 yrs) 115 (age ≥ 40 yrs)	+33% +92%
Women	40	45 (age < 40 yrs) 75 (age ≥ 40 yrs)	+12% +88%

Reference: Danielsson *et al*. [95].

## 10. Conclusions

Recent progress in laboratory medicine has provided us with novel possibilities for biomarker-based assessment of health risks related to excessive alcohol use and other factors of life style. The data gathered has also improved our understanding on the primary mechanisms of such problems. To date, approximately every sixth individual of the adult population in most Western countries drinks alcohol in excessive amounts. At the same time, half of the population suffers from being overweight. A more systematic use of biomarkers of alcohol consumption, including EtG and CDT or GT-CDT, improves the possibilities for early intervention in alcohol use disorders. Increased activities of serum liver-derived enzymes, ALT and GGT, are useful screening tools for liver affection but also prognostic indices of simultaneous extra-hepatic risks, such as metabolic syndrome, and cardio- or cerebrovascular events. GGT levels are linked with the status of oxidative stress, which is a key mechanism by which ethanol use promotes tissue injury. The presence of adiposity, unhealthy diet or smoking in alcohol consumers increases the risk for co-morbidities in a synergistic, age- and gender-dependent manner. In women and in those over 40 years of age, alcohol toxicity occurs at markedly lower levels of alcohol consumption. Epidemiological and biomarker-based evidence suggests that coffee consumption, in turn, may provide protection towards ethanol-induced oxidative stress. Biomarkers of inflammation, fibrogenesis and various specifically designed prognostic indices can provide additional value in the assessment of disease outcome in patients with alcohol-induced tissue damage. 

Future work should be aimed at establishing biomarker-based neural networks and prediction models for individual disease risk assessment. More accurate estimates of safe levels of ethanol consumption in different demographic categories are also needed. Correct definitions of biomarker normal limits should be the first step to be taken in this direction. 
